# Management of fibroids prior to in vitro fertilization/ intracytoplasmic sperm injection: A pragmatic approach

**DOI:** 10.4274/jtgga.galenos.2018.2018.0148

**Published:** 2019-02-26

**Authors:** Erdinç Sarıdoğan, Ertan Sarıdoğan

**Affiliations:** 1 Clinic of Obstetrics and Gynecology, University of Health Sciences, Ankara Zekai Tahir Burak Women’s Health Training and Research Hospital, Ankara, Turkey; 2 Department of Obstetrics and Gynecology, University College London Hospitals, London, United Kingdom

**Keywords:** Fibroids, leiomyoma, in vitro fertilization, assisted reproductive technology

## Abstract

Fibroids are relatively common in women undergoing in vitro fertilization (IVF) treatment due to their high prevalence in women. It is generally accepted that submucosal fibroids are deleterious to IVF outcomes and their removal is beneficial. Evidence from relatively low quality studies on the impact of intramural fibroids on IVF outcome is also suggestive of a detrimental impact. The majority of published studies included women with relatively small intramural fibroids and women with cavity-distorting fibroids were usually excluded, hence it is quite likely that the detected impact in the systematic reviews is an underestimation. Evidence of benefit is scarce for the removal of noncavity-distorting intramural fibroids. It is quite likely that numbers needed to treat for this purpose would be very high for small fibroids but lower for larger fibroids. This would need to be taken into account when decisions are made on myomectomy and potential benefits should be weighed against the associated morbidity, cost, and delay in fertility treatment. Whilst there is a need to perform prospective randomised studies in this field, a pragmatic approach that takes prognostic factors into account to estimate the magnitude of the possible impact of the fibroid(s) and potential benefit of removal is likely to lead to better reproductive outcomes.

## Introduction

Fibroids are common in women in their reproductive years and are frequently detected in women who are about to undergo treatment with assisted reproductive technologies (ART). Although many fibroids are completely harmless and have no clinical significance, 10-15% of white women and 30-40% of black women between the ages of 35 to 39 years have been found to have clinically relevant fibroids (uteri nine weeks gestation size or larger, at least one submucosal fibroid or at least one fibroid of ≥4 cm) (1). As a result, questions are inevitably raised by physicians and couples about the possible detrimental impact of fibroids on the planned ART or whether removal of fibroids would be expected to be beneficial in improving the ART outcome. The published literature on the impact of fibroids on fertility/fertility treatment outcome and potential benefit of fibroid removal is marred by a number of problems. The majority of studies are observational and are prone to selection bias. It is quite likely that women with larger and more ‘significant’ fibroids undergo surgery and are excluded from these studies. In addition, there are a large number of confounding parameters that are difficult to control in fibroid-related studies; fibroids come in all different numbers, sizes, and locations. Studies set out to diligently study the impact of intramural fibroids to ensure that uterine cavity distortion is excluded with a high quality or reliable test. This has resulted in exclusion of a large subgroup of women who have intramural fibroids with cavity distortion and the published systematic reviews do not provide a clear outcome analysis for this group. As a result, recommendations from professional organisations end up representing the opinion of the people who write them, sometimes with conflicting views even in the same document.

A number of meta-analyses since 2007 reported different conclusions despite mostly including the same studies (2,3,4,5,6,7). This is likely to be the result of differences in the methodology of reviews and inclusion/exclusion criteria that were used. In this article, the evidence from the published literature will be critically analysed in an attempt to provide guidance to physicians as to how fibroids can be managed in women undergoing in vitro fertilization (IVF)/intracytoplasmic sperm injection (ICSI) treatment.

### Evidence from Meta-Analyses

We will look at six major reviews that analysed the impact of fibroids on reproductive outcomes (2,3,4,5,6,7). Three of these reviews included studies that investigated the impact of all-type fibroids on both spontaneous pregnancies and IVF treatment outcomes (2,3,4), and the other three specifically addressed studies that assessed the impact of intramural fibroids that did not distort the uterine cavity on the outcome of IVF treatment (5,6,7).

Somigliana et al. (2) conducted a number of meta-analyses on the published literature related to fibroids and reproduction. In one of these, they assessed 16 articles on IVF outcomes and fibroids. Two studies, which included submucosal fibroids, showed that the presence of these fibroids significantly reduced pregnancy [odds ratio (OR): 0.3, 95% confidence interval (CI): 0.1-0.7] and delivery rates (OR: 0.3, 95% CI: 0.1-0.8). Intramural fibroids (seven studies) caused a small but significant detrimental impact of intramural fibroids on conception (OR: 0.8, 95% CI: 0.6-0.9) and delivery (OR: 0.7, 95% CI: 0.5-0.8) rates following IVF/ICSI treatment. Studies showed that subserosal or intramural/subserosal fibroids did not significantly reduce IVF/ICSI outcomes. They noted that the average diameter of fibroids in the included studies was rarely above 3 cm and that the detrimental impact emerging from the published articles may have been an underestimation of the real impact. They based this opinion on the finding that the negative impact was seen in women with fibroids >4 cm (8). Somigliana et al. (2) highlighted a nonrandomised comparative study by Bulletti et al. (9) who found higher cumulative clinical pregnancy (33% vs 15%) and delivery (25% vs 12%) rates after one to three cycles of IVF treatment in women who underwent myomectomy for intramural fibroids >5 cm compared with those who decided against myomectomy.

Klatsky et al. (3) included three studies on submucosal fibroids and IVF outcomes. This showed a significant reduction in implantation (OR: 0.39, 95% CI: 0.24-0.65) and clinical pregnancy rates (OR: 0.44, 95% CI: 0.28-0.70) and increase in miscarriage rates (OR: 3.89, 95% CI: 1.12-13.27). Nineteen studies compared IVF outcomes in women with intramural fibroids of 1-8 cm with those of controls without fibroids. Most studies included women with relatively small fibroids of 2-3 cm. The meta-analysis by Klatsky et al. (3) showed a significant decrease in implantation (OR: 0.79, 95% CI: 0.71-0.88) and clinical pregnancy rates (OR: 0.84, 95% CI: 0.74-0.95) and increase in miscarriage rates (OR: 1.82, 95% CI: 1.43-2.30). Klatsky et al. (3) did not analyse the impact of subserosal fibroids on IVF outcomes.

Pritts et al. (4) analysed 23 studies, which mostly gave IVF/ICSI related outcomes. Four of these studies on submucosal fibroids showed significantly reduced clinical pregnancy (OR: 0.36, 95% CI: 0.18-0.74), implantation (OR: 0.283, 95% CI: 0.12-0.65), and ongoing pregnancy/live birth rates (OR: 0.32, 95% CI: 0.12-0.85) and increased miscarriage rates (OR: 1.678, 95% CI: 1.37-2.05). Twelve studies that included outcomes related to intramural fibroids showed lower clinical pregnancy rate (OR: 0.81, 95% CI: 0.70-0.94), ongoing pregnancy/live birth (OR: 0.70, 95% CI: 0.58-0.85), and implantation rates (OR: 0.68, 95% CI: 0.59-0.80), and higher miscarriage rates (OR: 1.75, 95% CI: 1.23-2.49) compared with control women without fibroids. When only prospective studies or studies that assessed uterine cavity distortion with hysteroscopy or sonohysterography were included, the implantation rates remained significantly lower in women with intramural fibroids, but clinical pregnancy rates were no longer significantly different. Two studies that assessed the clinical pregnancy rates and one that gave the ongoing/live pregnancy rates showed that myomectomy for intramural fibroids did not improve the outcomes compared with controls with in situ fibroids. This review did not show a significant impact of subserosal fibroids.

Sunkara et al. (5) published an analysis of 19 studies on the impact of non-cavity distorting intramural fibroids on IVF outcomes. They found significant reductions in live birth rates (OR: 0.79, 95% CI: 0.70-0.88) and clinical pregnancy rates (OR: 0.85, 95% CI: 0.77-0.94) in women with fibroids compared with women without fibroids. Implantation and miscarriage rates were not statistically different. The studies included in this article had data from women with fibroids of 0.4-8.0 cm, the majority being less than 5 cm.

Metwally et al. (6) conducted a further analysis of the effect of intramural fibroids on ART treatment using published studies that included an aged-match control group, analysed intramural fibroids separately (not grouping them together with subserosal fibroids), and excluded submucosal fibroids by assessing the endometrial cavity using an objective method (hysteroscopy or sonohysterography). With this approach, no differences in live births, clinical pregnancy or miscarriage rates were found between women with and without fibroids. However, inclusion of studies with less strict criteria suggested lower clinical pregnancy rates (OR: 0.60, 95% CI: 0.42-0.87), whilst live birth and miscarriage rates were still similar. Importantly, four studies that gave the size of fibroids included women with fibroids sized of 5 cm or less.

Wang et al. (7) recently performed an updated meta-analysis of the impact of noncavity-distorting fibroids on the outcomes of IVF. The authors included 28 studies comprising 9189 IVF cycles, including the 19 studies included in the meta-analysis by Sunkara et al. (5). Seven of these were prospective trials and 23 studies controlled for compounding factors such as the woman’s age. This meta-analysis demonstrated significantly reduced clinical pregnancy [risk ratio (RR): 0.86, 95% CI: 0.80-0.93], live birth (RR: 0.81, 95% CI: 0.73-0.91) and implantation rates (RR: 0.90, 95% CI: 0.81-1.00) and increased miscarriage rates (RR: 1.27, 95% CI: 1.08-1.50). Separate analysis of prospective studies only and outcome of first cycle IVF confirmed the detrimental impact of noncavity-distorting fibroids on clinical pregnancy and live birth rates. 

It appears that, despite some degree of differences in the conclusions of these systematic reviews, the common finding is that the presence of fibroids has a detrimental impact on the outcome of IVF. It is generally accepted that submucosal fibroids do have a detrimental impact on fertility outcome. However, the quality of evidence to support this is weak and the significance of benefit of submucosal fibroid removal was brought into question in a Cochrane review (10).

### Importance of Fibroid Size

A common feature in the majority of studies is that they included only women with relatively small intramural fibroids, probably because women with larger fibroids were excluded and underwent a myomectomy. Hence, the published literature is very likely underestimating the impact of intramural fibroids, particularly larger fibroids. 

Only a few studies attempted to assess the impact of fibroid size. Oliveira et al. (8) found that a detrimental impact was seen in the presence of relatively larger fibroids. The clinical pregnancy rates were lower after IVF/ICSI in women with intramural or subserosal fibroids of 4.1-6.9 cm compared with women with no fibroids or fibroids ≤4 cm. There was no difference in pregnancy rates between the control group and women with fibroids ≤4 cm. Women with fibroids of ≥7 cm were excluded.

Another retrospective study of impact of fibroids that did not distort the cavity found that delivery rates were lower in the presence of fibroids >2.85 cm, whilst there was no detrimental impact in the presence of smaller fibroids (11).

A more recent retrospective matched cohort study showed that fibroids ≥30 mm had a deleterious effect on live birth rates, whereas this effect was not seen in the presence of fibroids <30 mm (12).

### Impact of Fibroid Removal

Evidence on the potential benefit of removal of fibroids prior to IVF/ICSI for women with fibroids is relatively scarce. A retrospective case controlled study of women with submucosal fibroids undergoing IVF using own or donated eggs showed that hysteroscopic or abdominal myomectomy for submucosal fibroids normalised the cycle outcomes. In this group of women, implantation and ongoing pregnancy rates were similar to the controls who had no fibroids, suggesting that the detrimental impact of submucosal fibroids is eliminated by fibroid removal (13).

A comparative non-randomised study assessed the potential benefit of myomectomy for intramural fibroids prior to IVF (10). One hundred sixty-eight women with at least one fibroid >5 cm were allowed to choose between myomectomy and expectant management prior to IVF. Submucosal fibroids were excluded. In the 84 women who had a myomectomy, clinical pregnancy (33% vs 15%, p<0.05) and delivery (25% vs 12%, p<0.05) rates were significantly better compared with the other 84 women who did not have surgery after one to three cycles of IVF treatment. 

Hysteroscopic myomectomy is a relatively safe procedure with minimal surgical morbidity. However, it can cause intrauterine adhesions, which could lead to a reduction in fertility and chances of success with fertility treatment. Special attention should be paid to treatment of multiple and large submucosal fibroids. Hysteroscopic removal of large fibroids is more challenging and multiple fibroid removal is more likely to cause intrauterine adhesions. 

Abdominal myomectomy is a major operation that can cause significant morbidity, especially in the presence of multiple and large fibroids. Potential long-term harm of postoperative pelvic adhesions on spontaneous conception is well recognised but the impact of myometrial trauma or intrauterine adhesions after abdominal myomectomy on IVF is less well recognised. At the same time, questions still remain on its effect on fertility and outcome of ART due to the absence of convincing evidence.

When an abdominal myomectomy is indicated, the potential benefits of the laparoscopic approach against open myomectomy have been well established (14). In comparison with traditional open myomectomy, the laparoscopic approach is associated with less postoperative pain and fever, and shorter hospital stay at the expense of longer operating times in a number of randomized clinical trials (15). Other potential advantages of the laparoscopic approach include a shorter recovery time with a quicker return to activities of daily living (16). 

There will be a need to delay pregnancy after myomectomy to allow the uterine wall to heal. This is relatively short after hysteroscopic myomectomy because it does not involve a myometrial incision, but needs to be long enough for the fibroid bed to ‘recover’ and be covered with endometrium. However, women are usually advised to avoid pregnancy for at least three months after abdominal myomectomies, resulting in delays in the planned IVF treatment. This may potentially be an issue for older women, particularly for those with reduced ovarian reserve. This delay may, however, be overcome by performing IVF before myomectomy and freezing the embryos for transfer after the recovery period. One potential problem with this approach is difficulties with access to the ovaries due to fibroids.

### Conclusions and a Pragmatic Approach to Management of Fibroids Prior to IVF/ICSI

There is overall consensus that submucosal fibroids have a detrimental impact on the chances of success with IVF/ICSI. Furthermore, there is some evidence of the benefit of myomectomy for submucosal fibroids to improve ART outcomes. For this reason, we make every effort to remove all submucosal fibroids in our practice. It is usually possible to remove all type 0 and I fibroids hysteroscopically (17). We administer gonadotropin releasing hormone treatment for 2-3 months when the fibroid is ≥4 cm to reduce the likelihood of two-stage procedures. We also aim to remove single type II submucosal fibroids <4 cm hysteroscopically; some 3-4 cm type II fibroids require a two- stage approach. For type II fibroids of ≥4 cm, we give serious consideration to abdominal myomectomy (laparoscopic when possible, open in the presence of numerous fibroids). We pay special attention to reducing the risk of intrauterine adhesions in the presence of multiple submucosal fibroids, including removal of fibroids on opposing walls in different sessions.

Subserosal fibroids are unlikely to have an impact on ART outcomes, except when they cause difficulties with ovarian access for egg collection. For this reason, the majority of subserosal fibroids are left alone during IVF cycles.

The management of noncavity-distorting intramural fibroids prior to IVF/ICSI is less straightforward. Current evidence suggests a detrimental impact of the presence of these fibroids; however, this is based on relatively low quality studies that show significant variability in selection criteria and outcome measures. This is not unexpected considering that fibroids come in different numbers, sizes, locations, and consistencies. There is a clear need to perform prospective randomised studies on this subject, but this is likely to be difficult due to a high number of confounding factors that would be difficult to stratify. 

A major problem with the published studies that were analysed in the meta-analyses is that they included women with relatively small intramural fibroids, probably because women with larger fibroids and those with fibroids that distort the cavity undergo myomectomy. Therefore, the real impact of these fibroids on IVF outcomes is likely to be larger. An additional problem is that there is a shortage of evidence regarding benefit of removing noncavity-distorting intramural fibroids. However, abdominal myomectomy (laparoscopic or open) is relatively frequently performed for these fibroids. It is likely that the numbers needed to treat (NNT) for this purpose would be lower for larger fibroids but very high for small fibroids. This point would need to be weighed against the associated morbidity, cost, and delay in treatment when decisions are made on myomectomy. In our practice, we take the number and size of fibroids, the overall size of the uterus, history of previous surgery, and ovarian accessibility into account when we counsel patients who have intramural fibroids that do not distort the cavity prior to IVF treatment. We try to avoid surgery in the presence of fibroids <5 cm when the uterine cavity is regular. We tend to offer surgery first to women with intramural fibroids ≥7 cm, but proceed with IVF treatment without surgery in the presence of fibroids of 5-6 cm in the first IVF attempt. We usually offer surgery for fibroids of 5-6 cm if the woman had one or two failed IVF attempts. This approach aims to keep the NNT as low as possible per additional pregnancy achieved.

If there are difficulties with ovarian accessibility due to fibroids, we prefer surgery before IVF. We usually wait for three months before proceeding with IVF postoperatively, but in older women with reduced ovarian reserve, we proceed with IVF earlier and freeze embryos for delayed transfer.
